# How Mothers and Childfree Women Redefine Fulfillment: A Comparative Study of Life and Marital Satisfaction in a Pronatalist Society

**DOI:** 10.3390/ijerph23030349

**Published:** 2026-03-10

**Authors:** Sinem Burcu Uğur, Nehir Yasan-Ak, Aylin Çiçekli, Seda Tan

**Affiliations:** 1 Department of Social Work, Manavgat Faculty of Social Sciences and Humanities, Akdeniz University, Antalya 07600, Türkiye; sinemburcu@akdeniz.edu.tr; 2 Department of Management Information Systems, Manavgat Faculty of Social Sciences and Humanities, Akdeniz University, Antalya 07600, Türkiye; 3 Department of Sociology, Faculty of Letters, Akdeniz University, Antalya 07070, Türkiye; aylincicekli@akdeniz.edu.tr; 4 Department of History, Faculty of Letters, Akdeniz University, Antalya 07070, Türkiye; sedatan@akdeniz.edu.tr

**Keywords:** voluntary childlessness, mothers, life satisfaction, marital satisfaction, employed women

## Abstract

**Highlights:**

**Public health relevance—How does this work relate to a public health issue?**

- Voluntary childlessness and motherhood were both associated with women’s mental well-being and social experiences in a pronatalist context.
- Qualitative findings indicated that pronatalist norms were linked to experiences of stigma, stress, and psychosocial strain among women, regardless of parental status.

**Public health significance—Why is this work of significance to public health?**

- This study found no significant differences in marital or life satisfaction between mothers and voluntarily childless women.
- Well-being outcomes appeared to be shaped less by parental status itself and more by the sociocultural context surrounding motherhood and childlessness.

**Public health implications—What are the key implications or messages for practitioners, policy makers and/or researchers in public health?**

- The findings highlight the relevance of considering diverse reproductive trajectories when addressing women’s well-being in pronatalist societies.
- The results point to the importance of addressing gendered expectations and social norms as contextual factors influencing women’s psychological and social health.

**Abstract:**

In pronatalist societies where motherhood remains symbolically central to feminine identity, women’s well-being is shaped by gendered expectations surrounding reproduction. Within such contexts, understanding how different reproductive trajectories relate to marital and life satisfaction becomes particularly important. This study compared the marital and life satisfaction of mothers and voluntarily childless women in Türkiye, a pronatalist society. A sequential explanatory mixed-method design was employed. Quantitative data were collected from 178 employed married women (31 voluntarily childless, 147 mothers) using standardized life and marital satisfaction scales. This was followed by in-depth interviews with 22 participants (11 from each group). The quantitative findings indicated no statistically significant differences in marital or life satisfaction between the two groups. However, qualitative analysis revealed that comparable experiences of satisfaction were constructed through distinct normative pathways. Mothers derived fulfillment from culturally validated maternal identities despite increased responsibilities, whereas childfree women constructed satisfaction around autonomy, relational equality, and deliberate ethical choice within a pronatalist context. While voluntary childlessness broadens the repertoire of feminine identities, motherhood remains a powerful symbolic reference point for both groups. Rather than signaling the erosion of pronatalist norms, the findings suggest their ongoing renegotiation within contemporary Turkish society. These dynamics underscore the importance of addressing role-based stigma and supporting diverse reproductive choices in efforts to promote women’s psychological well-being and social equity.

## 1. Introduction

There has been a trend towards low fertility over the last 50 years. One of the most important dynamics behind this trend is the increase in the number of couples who decide not to have children. Research shows that in late modern societies, parenthood has become a conscious choice rather than a natural consequence of marriage. Women often play a central role in this decision, given that childbearing and childcare expectations disproportionately shape women’s lives. As women become more empowered socioeconomically and their status in society improves, they gain the agency to make this decision. Consequently, the number of childless couples is rising slowly but steadily worldwide [1,2,3,4,5,6,7,8,9]. While voluntary childlessness rates have reached record levels, especially in developed regions such as North America and Western Europe [10,11,12], this preference can find supporters even in pronatalist societies that view motherhood as both a civic duty and a fundamental part of women’s feminine identity [13]. As voluntary childlessness becomes a widespread phenomenon worldwide, determining the effects of this choice on married women’s life and marital satisfaction is also gaining importance. Research on this subject is quite limited and existing studies are limited to the geographies where the phenomenon is widespread. Thus, this study was conducted in Türkiye, where the childfree preference has emerged as a new phenomenon and has pronatalist characteristics. In Türkiye, pronatalist orientations are sustained through strong cultural expectations and family-centered policy discourses that closely associate womanhood with motherhood and frame childbearing as a moral and social ideal [14,15,16]. Motherhood is commonly constructed around intensive and idealized caregiving norms that place primary responsibility for children’s emotional and developmental well-being on women [17].

Alongside these enduring norms, late modern transformations observed globally—such as rising levels of women’s education and labor force participation, increasing individualization, and heightened awareness of the emotional and material demands of parenting—have increasingly challenged traditional motherhood ideals. These shifts, as reflected in feminist debates associated with third-wave feminism since the 1990s, have drawn attention to the socially constructed nature of gender roles and the plurality of women’s life trajectories [10,18,19]. Within this perspective, motherhood is not rejected but reinterpreted as one possible identity among many, disentangled from fixed gender categories and patriarchal prescriptions. However, the extent to which such reinterpretations are realized varies across social and cultural contexts [20]. In Türkiye, these changes have unfolded unevenly, creating a context in which pronatalist norms remain influential, while alternative life-course trajectories, including voluntary childlessness, have become more visible. This coexistence of enduring pronatalist ideals and shifting life-course orientations shapes a complex decision-making environment for women’s reproductive choices. Within this context, childbearing remains institutionally and culturally embedded within marriage [21]. In Türkiye, the transition to parenthood is largely structured through marriage as its primary legitimate framework, and motherhood is normatively linked to marital status. For this reason, remaining childfree within marriage constitutes a more analytically visible departure from dominant pronatalist expectations than childlessness outside marriage, which represents a distinct social category shaped by different norms and life-course trajectories. Focusing on married women therefore allows for a clearer examination of how motherhood as a cultural norm is affirmed, negotiated, or reinterpreted within the very institutional setting where it is most strongly prescribed. Against this backdrop, the present study examines how preferences regarding parenthood—whether to have children or to remain childfree—are associated with the lives of married women in Türkiye.

This research focused on understanding the impact of the preference regarding parenthood (to have or not to have children) on the lives of married women; to this end, both mothers and childfree women were included in this study. Given that employment plays a significant role in shaping decisions about having children and childcare practices, this study specifically focused on working women. In pronatalist societies like Türkiye, where motherhood is strongly linked to female identity, understanding the psychological impact of childbearing choices is critical for developing effective public health interventions focused on mental well-being, marital stability, and life satisfaction among women.

### 1.1. The Child: The Main Actor in the Differentiated Experiences of Married Women

In previous studies conducted with married women, it was observed that both mothers and women who chose to remain childfree were generally satisfied with their lives and marriages [22,23,24]. However, both in these studies and in subsequent studies, it has been understood that motherhood and non-motherhood make a significant difference in women’s lives. The role of motherhood can be a source of personal fulfillment, pleasure, love, pride, satisfaction and joy. Through motherhood, women can gain a feminine and moral identity [25,26,27] and have access to broader social support networks. However, the cost of this role is that it can be associated with pain, feelings of inadequacy [28,29], vulnerability, helplessness, anger, hostility and frustration, as well as with a loss of leisure time, energy, and ultimately identity [19,26,27]. Motherhood can also undermine the marriage by placing the child above the marital relationship, particularly above the father, thereby damaging the close, intimate, fulfilling, and friendship-based relationship between spouses, leading the marriage into a deadlock [10,19].

On the other hand, voluntary childlessness offers married women freedom, economic and emotional autonomy, security [30,31,32], career opportunities, job satisfaction, a better financial position, and the chance to benefit from all kinds of opportunities for self-fulfillment [33,34]. The decision to “forgo” motherhood by deviating from accepted social norms can be associated with increased life satisfaction alongside reduced concerns about the future, as well as the possibility of having an egalitarian relationship, maintaining fulfilling relationships with partners, and preserving emotional and sexual energy [16,19,31,35,36,37]. However, the cost of the childfree choice, characterized by greater freedom, is the stigmatization of childfree women as selfish and the devaluation of their status. This stigmatization can range from perceiving childless women as individuals driven by pleasure to deeming them unreliable or even dangerous to society, thus devaluing them. For instance, during the 2024 U.S. presidential election, a candidate deliberately targeted their childless female opponent and her supporters by using the phrase “childless cat ladies.” This rhetoric was employed as a strategic approach to label the opponent as “anti-child and anti-family,” thereby alienating her. Especially in pronatalist societies, this choice can lead to women being perceived as unhappy and worthless, met with pity and sadness, and subject to discrimination and stigmatization [38,39,40], ultimately resulting in lower life satisfaction [32]. As revealed by the studies whose findings are discussed, both motherhood and being childfree come with rewards and costs. Despite the existence of a relatively extensive body of literature focusing on the advantages and disadvantages of the decision of whether or not to have children, studies examining this decision in terms of marital and life satisfaction are much more limited. To our knowledge, comparative evidence focusing simultaneously on marital and life satisfaction among mothers and voluntarily childfree women remains limited, particularly in pronatalist contexts.

### 1.2. Theoretical Framework of This Study

In this study, the impact of voluntary childlessness and parenthood on working women’s marital and life satisfaction is grounded in two main theoretical approaches that help explain how motherhood is socially constructed and how gendered expectations shape women’s experiences within marriage and everyday life. The first of these theoretical approaches is Bem’s gender schema theory [41]. According to this theory, motherhood, like other elements of identity, is a product of social construction. Women internalize motherhood as a feminine role, value, and responsibility through the socialization process that spans from childhood to adulthood. In patriarchal gender cultures, women come to attribute motherhood as their primary duty and the highest status they can achieve, and they are socialized to attain this position. From this perspective, motherhood represents a culturally privileged identity position that may be expected to align with socially valued understandings of femininity, whereas voluntary childfreedom may be perceived as less culturally endorsed within pronatalist contexts. In this sense, motherhood and childfreedom constitute identity-relevant positions that shape how women evaluate their lives and relationships. Within this framework, marital and life satisfaction can be understood as subjective reflections of how internalized gender schemas surrounding motherhood and non-motherhood are experienced and evaluated in everyday life. The second theoretical foundation of this study is second-wave feminism [42], which critiques the systemic sexism ingrained in society. This perspective rejects a single, homogeneous definition of womanhood and conceptualizes women’s identities—closely intertwined with motherhood—as socially constructed. It argues that caregiving, childrearing, and nurturing practices, which are not biologically determined, are transformed by patriarchal ideology into emotional and moral obligations closely associated with female identity. As a result, women are disproportionately confined to the domestic sphere and distanced from socially privileged positions. In the context of this theoretical framework, the purpose of this study is to understand the difference in marital and life satisfaction between voluntary childless women and mothers. Specifically, the present study proposed the following research questions:

1. What is the marital satisfaction level among voluntarily childless women and mothers?
2. What is the life satisfaction level among voluntary childless women and mothers?
3. Is there a difference in marital and life satisfaction between voluntary childless women and mothers?
4. How do voluntarily childless women and mothers differ in terms of marital satisfaction and life satisfaction experiences?

## 2. Methods

### 2.1. Research Design

This study employs a sequential explanatory mixed-method design [43] to investigate differences in marital and life satisfaction between childfree women and mothers. The quantitative phase was designed to provide a broad, descriptive overview of patterns in marital and life satisfaction rather than to produce population-representative or generalizable estimates. Standardized surveys were used to explore potential differences between the two groups and to situate this study within existing quantitative research. The subsequent qualitative phase constitutes the core analytical component of this study. Drawing on descriptive phenomenology, semi-structured interviews were conducted to explore how childfree women and mothers interpret and experience marital and life satisfaction in their everyday lives [44]. This qualitative approach allows for a nuanced understanding of meanings, perceptions, and relational dynamics that cannot be captured through survey data alone. By integrating these quantitative and qualitative strands, this study aims to contextualize exploratory quantitative findings and deepen their interpretation through qualitative insights, rather than to test hypotheses or make causal claims [45]. This mixed-method design aligns with this study’s theoretical emphasis on the social construction of gender roles and the diversity of women’s lived experiences, while also directly supporting this study’s research questions by combining comparative patterns with in-depth accounts of women’s subjective experiences.

### 2.2. Data Collection Procedure and Participants

The target population of this study consisted of employed, married women who identified themselves as either mothers or voluntarily childfree, with data collected from women living in Türkiye. The focus on married women was a deliberate analytical choice. In the Turkish sociocultural context, motherhood is normatively embedded within marriage, and childbearing outside marriage remains socially marginal. Consequently, voluntary childlessness becomes socially and symbolically meaningful primarily within marriage. Including unmarried women would have introduced a different social category rather than a comparable reproductive choice within the same institutional framework. Data were gathered between May and December 2023 from employed women who identified as either mothers or childfree women. For the quantitative component, a convenience sampling method was employed, which may limit the generalizability of the findings [45]. An online survey was administered via Google Forms, beginning with an informed consent statement outlining voluntary participation and confidentiality; only completed questionnaires were accepted. Of the 191 submissions, 13 were excluded because participants did not meet the inclusion criteria (being women, employed, and identifying as either mothers or childfree). A total of 178 participants were included in this study, with 31 (17.4%) voluntarily childless women and 147 (82.6%) mothers as seen in Table 1. The participants’ ages ranged from 24 to 59 years, with a mean age of 38.17 years (SD=6.86). The average number of years the participants had been working was 14.94 (SD=7.79), ranging from 1 to 39 years. In terms of marital duration, the mean length of marriage was 11.34 years (SD=7.79), ranging from 0.08 to 37 years. Regarding education levels, 66.9% of the participants held an associate or bachelor’s degree, 24.2% had a master’s degree, 9% had earned a doctorate (Ph.D.), and a smaller portion had completed high school (6.7%) or middle school (0.6%). Among the 141 participants who were mothers, the average number of children was 1.61 (SD=0.70), with participants reporting between 1 and 5 children. The ages of the children ranged from 1 month to 35 years, with a mean age of 9.70 years (SD=7.30).

For the qualitative component, a purposive snowball sampling technique was used to recruit participants with varied motherhood decisions. In total, 22 participants (11 mothers, 11 childfree women) contributed to semi-structured interviews, each lasting at least one hour and continuing until no new information emerged [46]. The demographic and social characteristics of the participants who were interviewed in-depth are presented in Table 2.

In the study population, there are 22 women actively working in their professional lives. The ages of these women range from the youngest at 25 to the oldest at 44, with an average age of 36.09. The education level among the participants is high, with a concentration at the bachelor’s degree level (36.6%). Bachelor’s degrees are followed by doctoral-level education at 23.3%. Except for one high school graduate, the other participants are understood to have completed a master’s degree. These women have, on average, been working for 10 years, and the majority hold high-status, stable, and insured professions, in line with their education levels. Of the 22 women, whose marriage durations range from 1 to 23 years, 11 are childfree, while the others either have one child (6 women) or two children (5 women). It was found that almost all women with children (except for one participant) have at least one child under 14 years old, meaning their children have not yet reached adolescence. This study received ethical approval from Akdeniz University’s Research Ethics Committee on 20 May 2023 (Approval no. 646869).

### 2.3. Data Analysis

This study employed both quantitative and qualitative analytic approaches, in keeping with the sequential explanatory mixed-method design. In the quantitative analysis, descriptive statistics were used to summarize participants’ characteristics, followed by an assessment of reliability for the Marital Satisfaction Scale (MSS) and the Satisfaction with Life Scale (SWLS) using Cronbach’s alpha. A One-Way Multivariate Analysis of Variance (MANOVA) was then conducted to examine potential differences in marital and life satisfaction between childfree women and mothers. MANOVA was chosen for its capacity to handle multiple dependent variables across groups while controlling for Type I error [47,48]. Sample size calculations via G*Power 3.1, using a Pillai’s V effect size of 0.0625, α=0.05, and power = 0.80, recommended a minimum of 158 participants; the final sample comprised 178 after exclusions. Before conducting the one-way MANOVA, all relevant assumptions were examined. The analysis included two continuous dependent variables—marital satisfaction and life satisfaction—and one categorical independent variable representing the reason for not having children, categorized as Childfree Women and Mothers. Observations were independent, with each participant belonging to only one category. The assumption of multivariate normality was assessed using the Kolmogorov–Smirnov one-sample test. Although the *p*-values for both the SWLS and MSS were 0.000, visual inspection of box plots and bivariate scatterplots showed no serious violations. Skewness and kurtosis values fell within the acceptable range (−3 to +3), and no univariate outliers were detected based on z-scores exceeding ±3.29. Multivariate outliers were assessed using Mahalanobis distance, and none were identified. A linear relationship was observed between each pair of dependent variables within each group. Levene’s Test confirmed homogeneity of variances (p>0.05 for both SWLS and MSS), and Box’s M test supported homogeneity of covariances (p=0.622). No multicollinearity was detected; the correlation between SWLS and MSS was 0.473, which is acceptable and indicates related yet distinct constructs. All assumptions for the one-way MANOVA were satisfied, and analyses were conducted using IBM SPSS 28.

For the qualitative component, content analysis of the semi-structured interviews was undertaken to explore in depth how childfree women and mothers perceive and experience marital and life satisfaction. Interview transcripts were systematically coded and organized into categories to identify emergent themes, with thick description used to capture the complexity of participants’ lived experiences. To enhance analytical rigor, multiple researchers reviewed and discussed the coding and thematic structure until consensus was reached [45]. Internal reliability was supported by presenting participants’ accounts verbatim, preserving fidelity to their original expressions. To strengthen transparency and credibility, the research process and analytic steps were documented in detail, enabling analytical coherence and facilitating future replication. Integration of the quantitative and qualitative findings is presented in line with this study’s objectives in the subsequent sections.

### 2.4. Data Collection Tools

The data for this study were collected using a survey form that consisted of three main sections: demographic information, the Marital Satisfaction Scale (MSS), and the Satisfaction with Life Scale (SWLS). Additionally, a semi-structured interview form was used for the qualitative phase. A detailed description of each tool is provided below.

#### 2.4.1. Personal Information Form

The personal information form collected demographic data from participants, including age, gender, educational status, employment status, profession, duration of marriage, number of children, and the age of children. This section was designed to gather basic background information relevant to understanding the context of participants’ responses.

#### 2.4.2. Marriage Satisfaction Scale (MSS)

The Marital Satisfaction Scale (MSS) was developed by [49] to measure marital satisfaction. It consists of 13 items, including 5 positive and 8 negative items, scored on a 5-point Likert scale ranging from 1 (“Not at all suitable for me”) to 5 (“Completely suitable for me”). The scale measures three subdimensions: Family, Sexuality, and Self. Higher scores on the scale indicate higher levels of marital satisfaction. The scale has demonstrated high internal consistency, with a Cronbach alpha value of 0.82 in this study. The subscale alpha values were 0.83 for Family, 0.81 for Sexuality, and 0.75 for Self.

#### 2.4.3. Satisfaction with Life Scale (SWLS)

The Satisfaction with Life Scale (SWLS) was originally developed by [50] to assess individuals’ overall satisfaction with their lives. The scale comprises five items, scored on a 7-point Likert scale ranging from 1 (“Strongly disagree”) to 7 (“Strongly agree”). The Turkish adaptation of the scale was conducted by [51], and it has been shown to have strong internal consistency, with a Cronbach alpha of 0.81 in the current study.

#### 2.4.4. Semi-Structured Interview Form

The interview questions were developed based on both the research objectives and the findings from the quantitative phase. The interview topics included motivations for remaining voluntarily childless or choosing to have children, the impact of these decisions on marital satisfaction, and participants’ perspectives on their life satisfaction. Sample questions from the interview form included: “Why did you choose to get married? How would you define marriage in your own words? What role do you think children play in marriage? How would you describe your decision to remain childfree, and what motivates you to continue with this choice? In what ways has having children affected your marital relationship? How is your choice of childfree perceived by others, such as your spouse, family, or friends?”. These questions were designed to encourage participants to freely express their unique experiences and perspectives regarding motherhood choices and their impact on life and marital satisfaction. The form was reviewed by two experts for clarity, scope, and applicability. A pilot study was conducted with four participants, and no further revisions were deemed necessary.

## 3. Results

### 3.1. Quantitative Findings

This section presents the quantitative results (see Table 3) addressing the marital and life satisfaction levels of voluntarily childless women and mothers, as well as potential differences between the two groups. Childfree women reported a high mean marital satisfaction score of 53.35 (SD=9.84), while mothers had a similar mean score of 52.50 (SD=9.29). For life satisfaction, mothers reported a higher mean (M=23.79,SD=7.05) than childfree women (M=21.61,SD=6.09). These results suggest minimal differences in marital satisfaction between the groups, while mothers reported slightly higher life satisfaction.

The results of the MANOVA, presented in Table 4, indicated that there was no statistically significant difference in marital and life satisfaction based on motherhood status, F(2,175)=2.241, p>0.05, Pillai’s Trace =0.025. Due to the unequal sample sizes between groups, Pillai’s Trace was used as the most robust test statistic. Furthermore, there was no significant effect of motherhood status on marital satisfaction, F(1,176)=0.211, p=0.647, partial $\eta^{2}=0.001$, and on life satisfaction, F(1,176)=2.547, p=0.112, partial $\eta^{2}=0.014$, when the univariate between-subjects ANOVA effects were assessed. Post-hoc tests were not conducted because there were fewer than three groups.

### 3.2. Qualitative Findings

This research was conducted within the framework of the main research question: “How do marital and life satisfaction differ between women with children and those who choose to remain childless, depending on the variable of having children?” Interview questions prepared in line with this core problem were posed to the participants, and the responses were analyzed in detail for each question under the corresponding sub-problems. The analysis revealed three interrelated themes: (a) perception of marriage and family, (b) experiences of motherhood and childfreedom, and (c) mutual perceptions of childbearing and childfreedom.

#### 3.2.1. Theme 1: Childfree Women’s and Mothers’ Perception of Marriage and Family

When childfree women and mothers were asked how they define marriage and family, and what these concepts mean to them, a common definition emerged from both groups: *“Marriage is a companionship in a loving home/life/journey.”* This prevalent definition among the participants also reflects their reasons for getting married. It was observed that both mothers and childfree women primarily decide to marry with the motivation of *“sharing a life with the person they love”* and *“creating the warm family environment they long for.”* Additionally, a significant portion of the participants (n=7) stated that they perceived marriage as a social norm in Turkish culture and felt obligated to conform to it. As one participant pointed out, societal expectations played a decisive role in her decision to marry: *“Society says that if you want to share your life with someone, you must get married”* (P2, childfree). Women from both groups tended to define marriage in a positive light, emphasizing the emotional bond and shared life it provides. However, a smaller number of participants from both groups pointed out that marriage can also function as an institutional arrangement involving a large extended family and limiting personal privacy. This view was more commonly expressed among childfree women, who noted that these dynamics can occasionally make the relationship more challenging—a reality rarely depicted in the idealized portrayals of romantic relationships:

*“Hollywood romances end at the wedding, as if marriage were the final triumph of love. Yet real marriage is not a private duet but a crowded stage—where in-laws, family histories, and tangled obligations all take their place. To sustain it means not only loving one person, but learning to navigate an entire web of relationships.”* (P4, childfree)

Following the analysis of participants’ definitions of marriage, an examination of their conceptualizations of family reveals parallels between the two concepts. The majority of both mothers and childfree women defined family as *“a space of unconditional love, healthy communication, peace, and security.”* Some participants in both groups also described family as a structure grounded in mutual responsibilities, where membership entails fulfilling shared obligations. Notably, a considerable number of participants (n=6) defined family as a unit composed of a mother, father, and children, reflecting the traditional nuclear family model. While this definition appeared in both groups, it was significantly more prevalent among mothers.

Although both groups described marriage and family in largely affective terms—companionship, security, shared life—their conceptualizations diverged sharply regarding the necessity of children within the structure of family and marriage. For most mothers (n=10), children constituted the ontological core of family life and were perceived as the natural continuation—and completion—of marriage. In this view, having children was framed as both a personal and socially expected outcome, functioning simultaneously as a source of emotional fulfillment and a marker of marital identity (*“married-with-children”*). In this view, the child was not only a bond that strengthened family unity but also a defining element that legitimized and completed the family structure. While traces of the dominant “child-centered” family model appeared even among a few childfree participants, the overall pattern differed markedly from that of mothers. Rather than positioning children as the core of family life, childfree women redefined the child’s status within the family framework. For most, children were not a prerequisite for constituting a family; instead, family was grounded in emotional intimacy, shared life, and relational bonds. In some cases, these bonds extended to pets, described as legitimate family members. A childfree participant also strongly emphasized this perspective, highlighting that the sense of family is not necessarily tied to parenthood but rather to the emotional bonds formed with those we care for:

*“For example, I have a dog, and he is a member of our family. Just as you care for a child, we provide him with the same level of love and care. In that sense, I can define family as an environment made up of individuals and living beings with whom we can feel peaceful.”* (P3, childfree)

For these women, children were neither an indispensable condition for sustaining family or marital bonds nor a necessary source of personal meaning. In other words, they questioned the assumption that fulfillment must derive from motherhood. Remaining childfree was framed not as absence, but as a deliberate and self-determined choice aligned with personal priorities, autonomy, and alternative life projects. Within this perspective, fulfillment does not require dedicating oneself to a child; rather, it may emerge through partnerships, pets, professional aspirations, or personal growth. As P8 emphasized:

*“Just because I haven’t dedicated my life to someone—a child, to be specific—doesn’t mean I am irresponsible. On the contrary, I have devoted my life to myself and to science.”*

In sum, although both groups described marriage and family in affective terms, they diverged fundamentally in how they positioned children within these institutions. For mothers, children constituted the core of family and marital meaning, whereas childfree women decoupled family from parenthood.

#### 3.2.2. Theme 2: Experiences of Motherhood and Being Childfree

Across interviews, motherhood was consistently described as a profound restructuring of their identities, relationships, and everyday priorities. With the birth of a child, personal and marital identities were often reoriented around caregiving. The maternal role increasingly eclipsed professional, social, and spousal aspects of the self. Mothers also emphasized a marked increase in daily responsibilities and workload associated with childcare and domestic labor. As one participant noted:

*“Child has eclipsed every other role in my life—academic, spouse, daughter, sibling—leaving me defined above all as a caregiver.”* (P13, mother)

Within marital relationships in particular, this restructuring frequently took the form of a paradox. For the vast majority of mothers, having children brought spouses closer in their roles as parents. Shared responsibility and joint caregiving often strengthened their sense of partnership as co-parents. At the same time, however, this strengthening of the parental bond frequently coincided with a weakening of romantic partnership and sexual intimacy. This tension was described as particularly pronounced during the early years of a child’s life, when fatigue, sleep deprivation, diminished privacy, and unequal distribution of childcare intensified emotional distance and conflict. As P19 expressed:

*“Motherhood restricted my freedom while my husband’s remained largely intact. My exhaustion and frustration were often unseen or dismissed, leading to blame, emotional distance, and conflict—yet also greater tolerance for the child’s sake.”*

As children grew older, additional sources of conflict emerged. Differences in childcare and upbringing approaches between spouses often became a recurring point of tension. When partners held distinct beliefs and values regarding parenting—each grounded in their own convictions—these conflicting perspectives sometimes translated into disagreements and relational strain.

Motherhood also reshaped women’s relationships within their broader social environment. For many participants, becoming a mother marked a symbolic transition from being primarily positioned as a wife or daughter-in-law to being recognized as a mother within the extended family. This shift conferred social legitimacy not only upon their position as women within the family but also upon their marriages. The move from being *“just a couple”* to being acknowledged as a *“real family”*—particularly by parents and in-laws—signified an elevation in both personal and relational status. This increased recognition often contributed to warmer and more accepting relationships. At the same time, the arrival of a child introduced practical changes. As parents and in-laws became involved in childcare, everyday interaction intensified, fostering closer ties through shared responsibility, caregiving assistance, emotional support, and gratitude. In this way, relationships were strengthened both through symbolic recognition and through sustained daily involvement. However, for some participants, motherhood also generated tension within extended family relationships. Expectations surrounding childcare—particularly from parents and in-laws—occasionally became sources of disagreement and strain. Differences in beliefs about *“proper”* caregiving and childrearing practices sometimes escalated into criticism of the mothers’ parenting, intensifying relational conflict. In addition, a few participants reported weakened ties with family members who were either unwilling to participate in grandchild care or who expressed resentment toward those more actively involved. Such dynamics further complicated intergenerational relationships. Beyond extended family relations, motherhood significantly transformed women’s social networks. Many mothers reported that their friendships increasingly revolved around their children. Pre-existing friendships, especially with those without children, sometimes weakened, while new relationships formed with other mothers whose children were of similar ages.

In addition to reshaping relational dynamics, motherhood also transformed women’s economic lives. Economically, all participating mothers described a reorientation of financial priorities toward their children’s needs, with increasing expenses as children grew older. Although this shift did not always result in severe financial hardship, several participants reported experiencing financial strain or heightened economic pressure at certain stages.

Beyond relational and economic domains, participants described motherhood as a transformative yet demanding life experience. While it fostered emotional maturity and an expanded capacity for empathy, contributing to a deeper sense of fulfillment, it also entailed significant self-sacrifice, ongoing responsibility, and the postponement of personal aspirations. For some women, this postponement was associated with stress, feelings of inadequacy, perceived failure, and emotional depletion. As one mother reflected:

*“Motherhood is “an exhausting journey,” yet “the feeling of happiness outweighs the feeling of exhaustion.”* (P14, mother)

Consistent with these accounts of strain and fulfillment, nearly all participants stated that they would choose motherhood again if given the chance. Despite acknowledging sacrifice and exhaustion, they framed it as a demanding yet profoundly fulfilling life experience.

In contrast to mothers’ accounts of restructuring and sacrifice, childfree participants described continuity rather than transformation in their lives. Across interviews, remaining childfree was associated with the preservation of marital stability, relational harmony, and flexibility in organizing personal and professional priorities.

Within their marriages, most participants emphasized that remaining childfree did not undermine marital stability. With the exception of one participant who described relational strain and another who anticipated potential future tension due to patriarchal expectations surrounding parenthood, the majority characterized their marriages as stable and largely unaffected by the absence of children. While they did not explicitly portray their sexual lives in emphatic terms, none attributed relational strain or diminished intimacy to remaining childfree. Several participants suggested that having a child, rather than not having one, might have introduced tension into their relationships.

Beyond marital dynamics, childfree participants described noticeable differences within their social relationships. Within their close social environment—particularly relationships with parents and in-laws—pressure and questioning were common. For many, this decision was treated not as a settled choice but as an open issue—something to be reconsidered, negotiated, or eventually reversed. Childlessness was often perceived as temporary, problematic, or likely to result in future regret. Some women reported being viewed as incomplete or unsuccessful despite professional achievements. In contrast, friendships were described as largely unaffected by this decision, with many participants noting that their social circles were supportive and accepting of their choice.

Economically, participants consistently associated childfreedom with reduced financial burden and greater flexibility. Freed from the long-term economic responsibilities of childrearing, they described having increased control over spending, saving, and career-related investments.

Beyond relational and economic considerations, childfreedom was closely tied to autonomy and self-direction. Many women framed their decision as enabling them to prioritize personal aspirations, professional ambitions, and self-development. This autonomy was frequently linked to professional advancement, with several participants explicitly stating that the ability to allocate time, energy, and financial resources toward career goals enabled them to perform more successfully in their careers. As one participant articulated:

*“My life revolves around my ambitions, not obligation. Free from parental constraints, every pursuit—whether academia, sport, or dance—is entirely my choice.”* (P4, childfree)

Despite this emphasis on autonomy, a nuanced ambivalence was also present. Some participants acknowledged that motherhood might offer experiences of emotional richness absent from their lives. However, this reflection did not amount to regret. When asked whether they would reconsider their decision, the majority indicated that they would make the same choice under the same circumstances, though a few expressed openness to motherhood if broader structural and personal conditions were substantially different.

#### 3.2.3. Theme 3: Mutual Perceptions of Childbearing and Childfreedom

In this theme, participants evaluated women from the opposite group. By defining the motivations, responsibilities, and perceived consequences associated with women in the opposite group, participants indirectly illuminated how they situated their own decisions in relation to those portrayals. Across accounts, notions such as responsibility, morality, sacrifice, autonomy, and emotional fulfillment structured these evaluations, although the meanings attributed to these concepts diverged markedly between mothers and childfree women.

From the perspective of many mothers, childfreedom was interpreted as a conscious distancing from the responsibilities associated with parenthood. These participants often suggested that childfree women preferred to avoid the economic pressures, relational strains, career sacrifices, and profound lifestyle transformations that accompany prioritizing a child. In some accounts, childfreedom was interpreted not only as avoidance but also as an implicit acknowledgment of lacking the capacity or willingness to meet the demands of motherhood. Yet this interpretation did not uniformly translate into moral condemnation. On the contrary, several mothers expressed respect for women who deliberately chose not to become mothers, emphasizing that such a decision required self-awareness and careful deliberation. One participant even described childfreedom as ethically defensible in a world marked by uncertainty and structural instability. As one participant noted:

*“Childfree women often prioritize personal development, consciously rejecting the responsibility of parenthood. Their decision reflects deep awareness, careful deliberation, and a clear weighing of pros and cons.”* (P21, mother)

In several accounts, remaining childfree was even interpreted as a form of resistance to societal expectations surrounding motherhood. At the same time, a number of mothers associated childfreedom with emotional deprivation, suggesting that those who do not experience motherhood might be missing one of life’s most profound forms of joy and transformation. In a smaller subset of accounts, childlessness was even linked to psychological deficiency or the prioritization of traditionally “masculine” traits such as career ambition over caregiving.

In contrast, childfree participants interpreted responsibility in ways that differed markedly from the mothers’ accounts. Although a small number of mothers (n=2) interpreted voluntary childlessness as avoidance of parental responsibility, the majority of childfree women articulated an alternative understanding. For them, responsibility did not begin with giving birth. Rather, it began with ensuring that one possesses the necessary resources—time, energy, financial stability, emotional readiness, and genuine desire—to meet the long-term obligations of parenthood. In this view, bringing a child into the world was considered responsible only when undertaken with sufficient preparedness and conscious commitment. As P8 noted, parenthood could be deeply admirable when it involved deliberate commitment rather than automatic conformity to social expectations:

*“Bringing a person into the world and raising them feels like one of the highest achievements a person can reach… Being able to raise a self-sufficient individual—these are very valuable qualities.”* (P8, childfree)

Several childfree women explicitly stated that they admired mothers who embraced the burdens of caregiving and fulfilled parental responsibilities with commitment and sacrifice. Such mothers were regarded as selfless—particularly within a patriarchal context in which caregiving disproportionately falls on women. By contrast, these participants sharply criticized forms of parenthood they perceived as unreflective or instrumental. Having children primarily to conform to marital expectations, avoid loneliness, secure care in old age, or stabilize troubled relationships was described as irresponsible and ethically problematic. Against this backdrop, refraining from parenthood was framed not as an escape from responsibility but as its inverse: a self-aware and morally accountable stance. In this view, remaining childfree could function as a protective and even compassionate act—an ethical refusal to bring a child into conditions perceived as unstable, unjust, or insufficiently supportive. As P9 articulated, refraining from motherhood was understood not as lack of love for children, but as an expression of care:

*“I believe that by not bringing my children into the world, I am protecting them even more… perhaps it’s because I love them.”* (P9, childfree)

Even when personal comfort was acknowledged, childfreedom was more frequently framed as a carefully considered stance than as simple withdrawal from the demands of parenthood.

## 4. Discussion

### 4.1. Interpreting Similar Satisfaction in Divergent Life Paths

In the explanatory quantitative phase, no statistically significant differences were found between mothers and childfree women in terms of marital or life satisfaction. Both groups reported high marital satisfaction and moderate life satisfaction. Prior research presents mixed findings, with some studies reporting similarity between parents and childfree individuals [52,53,54] others identifying small negative effects of parenthood on women’s life satisfaction [29,55,56], and some suggesting slightly higher satisfaction among voluntarily childfree women in specific domains [57,58,59,60]. Given this variability, the absence of significant differences in the present study neither confirms nor contradicts the broader literature. Rather, it raises a more interpretive question: how are comparable satisfaction levels produced through distinct life trajectories?

The qualitative phase was designed precisely to address this interpretive gap. Drawing on established research identifying economic stability, education, urban residence, autonomy, relationship quality, health, and perceived control as key indicators of life satisfaction [23,29,61,62], we used these domains as interpretive lenses rather than as variables to be tested for presence or absence. Although the women in our sample shared structural advantages—being urban, highly educated, economically active, and married—the analysis focused on how the presence or absence of children reshaped the experience and prioritization of these domains. In other words, variation emerged not at the level of structural resources, but at the level of how motherhood or childfree status reconfigured autonomy, relational dynamics, social participation, economic arrangements, and perceived control over life decisions. The qualitative findings therefore illuminate how women defined satisfaction in their own terms within otherwise comparable life conditions.

The qualitative findings show that overall satisfaction can converge across groups because the sources of satisfaction differ. For mothers, fulfillment was often anchored in the emotional and identity-related meanings of motherhood, alongside marital partnership and family continuity, even as participants described increased responsibility, time scarcity, and work–family tensions. This ambivalent structure of reward and constraint parallels scholarship describing motherhood as both emotionally meaningful and structurally demanding [63,64,65,66]. For childfree women, satisfaction was more frequently constructed around autonomy, continuity in career and social life, relational equality, and control over time and resources, despite navigating normative expectations surrounding motherhood. Such narratives align with research suggesting that voluntary childlessness can be associated with heightened autonomy and self-determination, particularly among educated and economically active women [19,31,35,36]. These narratives correspond to established dimensions of life satisfaction—such as autonomy, relational quality, and perceived control [29,61]—yet they demonstrate that such dimensions are configured differently depending on reproductive choices. In other words, the quantitative similarity reflects not identical experiences but different pathways through which women negotiate fulfillment within the same sociocultural environment. Within sociocultural environments such as Türkiye, where reproductive roles remain closely intertwined with gender identity, marital expectations, and culturally privileged notions of femininity, recognizing that both mothers and women who choose not to become mothers construct fulfillment through distinct pathways becomes particularly meaningful. Rather than determining which group reports higher levels of satisfaction, this study shifts the focus toward how satisfaction is constructed and negotiated within a shared pronatalist cultural framework. By integrating qualitative analysis with gender schema theory and second-wave feminist critiques, the findings demonstrate that similar levels of satisfaction may emerge from structurally and symbolically distinct pathways. In this sense, this study contributes not by resolving quantitative inconsistencies in the literature, but by illuminating the normative and identity-based processes through which reproductive choices acquire meaning.

### 4.2. Marriage, Family, and the Negotiation of Motherhood as a Cultural Norm

Participants’ accounts of marriage and family were broadly aligned with the institutional organization of family life in the Turkish sociocultural context, where marriage commonly functions as the primary framework for family formation and parenthood. Across both groups, marriage was often described as an expected life-course transition and the conventional foundation of family life. However, this convergence at the institutional level did not translate into uniform meanings attached to marriage and family. A central contribution of this study lies in how marriage and family are conceptualized and how the child is positioned within these meanings. Across both groups, participants articulated definitions of marriage and family that closely resonate with dominant relational norms in Turkish society. Marriage was framed as a space of emotional intimacy, shared responsibility, and mutual commitment, while family was associated with unconditional support and moral obligation. Such convergence suggests that both mothers and childfree women operate within a shared normative framework of *“ideal”* womanhood and partnership. This alignment reflects what gender schema theory describes as the internalization of culturally structured expectations that shape how femininity and relational roles are cognitively organized [41]. However, it is important to note that the relational norms articulated by participants—emotional intimacy, mutual commitment, and shared responsibility—may themselves reflect broader transformations in urban middle-class marriage ideals in Türkiye. Historically shaped by kinship alliances and extended family structures, marriage in contemporary urban contexts has increasingly been redefined around companionate partnership and emotional fulfillment [67]. Given that the present sample consists primarily of educated, economically active, urban women, the convergence observed here may represent not timeless cultural norms, but the internalization of a relatively recent and class-inflected transformation in marital expectations.

The divergence emerges not at the level of marriage itself, but in the perceived necessity and centrality of children within the family unit. Among mothers, children were frequently described as the natural and expected extension of marriage. Motherhood was positioned as both identity-affirming and emotionally fulfilling, reinforcing the longstanding cultural intertwining of womanhood and caregiving documented in feminist scholarship [26,68,69]. In pronatalist contexts, where reproduction is symbolically linked to moral adulthood and social legitimacy, motherhood is often framed not merely as a personal choice but as a normative milestone [70,71,72,73,74]. The narratives of participating mothers reflect this schema: even when acknowledging the burdens of intensive caregiving, motherhood remained central to their understanding of a complete family and a meaningful feminine identity. In this sense, the findings illustrate the durability of gendered expectations that second-wave feminism critiqued as transforming socially constructed caregiving roles into moral obligations [42].

In contrast, childfree women articulated a more flexible understanding of family, decentering reproduction from its definition and emphasizing chosen bonds, relational equality, and shared life projects. This perspective resonates with feminist arguments that family structures and gender roles are neither fixed nor biologically determined, but socially produced and therefore open to reinterpretation. Yet this reinterpretation does not amount to a wholesale rejection of motherhood. Rather, childfree women frequently framed motherhood as an intensive and ethically demanding role—one that requires total responsibility and should be undertaken only under conditions of full commitment. In doing so, they simultaneously challenge the inevitability of motherhood while preserving its normative gravity. In a pronatalist context such as Türkiye, where motherhood remains symbolically central to femininity [71] and reproduction is normatively embedded in marital expectations both affirmation and deviation are structured by the same cultural schema.

These patterns suggest that the distinction between conformity and resistance is less clear-cut than often assumed. Instead of signaling the erosion of pronatalist norms, the findings point to an ongoing negotiation of gender schemas within the Turkish pronatalist context, where marriage, family formation, and motherhood continue to operate as culturally structured expectations. Mothers appear to affirm and inhabit dominant maternal ideals, whereas childfree women reposition these ideals within a framework of autonomy and choice. In both cases, gendered expectations surrounding motherhood remain symbolically powerful, but are interpreted and enacted differently. Thus, this study demonstrates not the disappearance of traditional norms, but their reconfiguration within late modern conditions where identity, choice, and obligation intersect.

A striking finding concerns the moral positioning adopted by childfree women. While they emphasize the costs of motherhood, they nonetheless frame it as an intensive and ethically demanding commitment—one that must either be fully embraced or consciously declined. This framing reflects the continued influence of gender schemas that equate motherhood with comprehensive caregiving and self-sacrifice. As gender schema theory suggests [41], culturally embedded expectations shape not only behavior but also evaluative standards. Even when women opt out of motherhood, they may continue to evaluate it through dominant normative criteria. Thus, rather than rejecting maternal norms outright, childfree women reinterpret them within a discourse of autonomy and responsible choice. Motherhood is recast as a voluntary, high-stakes decision in a context where fertility is controllable and individual self-realization is socially valued.

At the same time, this reinterpretation reveals the ambivalent power of pronatalist expectations. Many participants indicated that they might reconsider their decision under different life conditions, suggesting that motherhood remains symbolically meaningful even when not enacted. The insistence that motherhood must be performed *“properly”* illustrates how normative intensity is preserved. In this sense, gendered expectations are not dismantled but renegotiated—shifted from an unquestioned destiny to a morally evaluated choice.

The negotiation of gender norms becomes even more visible in how the two groups evaluate one another. Among some mothers, childlessness was associated with selfishness, emotional deficiency, or deviation from feminine ideals. Such perceptions align with research documenting the stigmatization of married but childless women, who are often required to justify their reproductive choices [26,75]. These attitudes indicate that pronatalist schemas continue to define the boundaries of legitimate womanhood. At the same time, even when childlessness was acknowledged as a conscious and legitimate choice, it was frequently interpreted through the lens of normative motherhood expectations. Some mothers suggested that childfree women had realistically recognized that they might not fulfill the intensive demands of ideal motherhood—whether due to career priorities, personal aspirations, or a perceived lack of maternal inclination. In this framing, voluntary childlessness is not positioned outside the normative framework but rather within it: it becomes a rational decision taken by those who assess themselves as unable or unwilling to embody culturally prescribed standards of total caregiving and self-sacrifice. Thus, even expressions of respect toward childfree women may reproduce the same gendered schema that defines *“proper”* motherhood as the benchmark against which women evaluate themselves and others. However, childfree women also engaged in moral positioning. In response to accusations of selfishness, some reframed their decision as more responsible, suggesting that bringing a child into the world without sufficient emotional or material readiness could itself be selfish. This strategy—previously identified in studies of voluntary childlessness [76]—reverses the moral hierarchy without escaping it. Rather than dissolving the normative framework, it reassigns moral superiority within it. Together, these dynamics illustrate that both groups actively negotiate gendered expectations. Mothers affirm motherhood as a culturally validated source of identity, while childfree women reconfigure it as a deliberate and ethically scrutinized choice. In this way, pronatalist norms are neither simply upheld nor rejected, but continually reworked in everyday life.

### 4.3. Limitations and Further Studies

While this study offers insight into how mothers and voluntarily childfree women negotiate marital and life satisfaction within a pronatalist context, several limitations should be acknowledged. First, the quantitative component relied on non-probability sampling and does not represent the broader population. Accordingly, the statistical findings should be interpreted as exploratory and contextual rather than generalizable. Future research employing representative samples would strengthen empirical claims regarding satisfaction differences across reproductive groups. Second, this study did not distinguish between voluntary and involuntary motherhood, which may mask important variations in how parenthood relates to well-being. Similarly, the developmental stage of children was not systematically examined, despite evidence that parenting demands fluctuate across the life course. Third, the sample consisted primarily of urban, highly educated, economically active married women. This homogeneity introduces a sampling bias that may have influenced the findings, particularly the convergence observed in marital and life satisfaction levels. Women with greater structural resources may experience higher autonomy, relational stability, and economic security, which could buffer potential differences between mothers and childfree women. Therefore, the findings cannot be assumed to reflect the experiences of women in rural areas, different socioeconomic contexts, or non-marital partnerships. Fourth, reliance on self-reported data introduces the possibility of social desirability bias, particularly in discussions of family, sexuality, and socially sensitive gender roles. Future studies might incorporate dyadic designs, longitudinal methods, or alternative data collection strategies to better capture relational and temporal dynamics. Expanding research across diverse demographic contexts and employing longitudinal approaches would deepen understanding of how life satisfaction evolves over time in both parental and childfree trajectories. Additionally, future research could extend the analysis to consciously childless men and single women in order to explore how gender and marital status intersect with reproductive decision-making. Including these groups would enable a more comprehensive understanding of voluntary childlessness within pronatalist societies.

## 5. Conclusions

This study demonstrates that similar levels of marital and life satisfaction among mothers and voluntarily childfree women do not indicate identical experiences, but rather distinct pathways of negotiating fulfillment within a shared pronatalist cultural framework. Mothers derive satisfaction from inhabiting culturally validated maternal identities, even while navigating structural and emotional constraints. Childfree women, in turn, construct fulfillment through autonomy, relational equality, and self-realization while continuing to engage with the normative weight of motherhood. The findings therefore highlight not a simple opposition between conformity and resistance, but an ongoing negotiation of gender schemas within Turkish society. Drawing on gender schema theory and second-wave feminist critiques, this study shows how motherhood remains a powerful symbolic category—even when declined—and how both groups reinterpret, rather than fully escape, culturally embedded expectations. Within this context, the absence of statistically significant differences in life satisfaction between the two groups can be understood as reflecting the adaptive capacity of women to construct meaning within structurally different but culturally interconnected life trajectories. The findings do not simply confirm or contradict prior research; rather, they suggest that understanding women’s well-being in pronatalist societies cannot be adequately captured through cross-group satisfaction comparisons alone. Instead, this study points to the importance of examining the distinct meaning-making processes and identity negotiations through which women construct fulfillment within normative gender regimes. This is particularly relevant in pronatalist settings such as Türkiye, where motherhood continues to operate as a culturally privileged marker of femininity and marital legitimacy, shaping both the enactment and the questioning of reproductive roles. From a research perspective, these findings encourage greater attention to interpretive and context-sensitive approaches that move beyond outcome differences to explore experiential pathways of well-being. From a practice perspective, the findings suggest that supporting women’s well-being in pronatalist societies requires acknowledging that motherhood is not the only meaningful life trajectory. This study also shows that mothers themselves may experience pressure to meet intensive motherhood ideals that generate substantial emotional and practical demands. Counseling practices can therefore help women navigate both the expectations attached to motherhood and the tensions surrounding non-motherhood. Workplace environments should avoid treating motherhood as a universal life course while also remaining attentive to the caregiving burdens often associated with maternal roles. At the policy level, recognizing voluntary childlessness as a legitimate life trajectory and addressing the normative and structural demands placed on women across reproductive pathways may contribute to supporting women’s psychological well-being.

## Figures and Tables

**Table 1:** Demographic characteristics of the quantitative data (N=178).

Variable	*f*	*%*
Voluntarily childless	31	17.4
Mothers	147	82.6
Education		
Middle school	1	0.6
High school	12	6.7
Associate/Bachelor’s degree	106	66.9
M.S.	43	24.2
Ph.D.	16	9.0
	***M***	***SD***
Age	38.17	6.86
Working year	14.94	7.79
Marriage year	11.34	7.79
Number of kids (n=141)	1.61	0.70
Kids age	9.70	7.30

Note. *f*: Frequency; %: Percentage; *M*: Mean; SD: Standard Deviation.

**Table 2:** Demographic characteristics of the qualitative data.

Part.	Age	Edu.	Profession	Work Yr	Marr. Yr	Mother or Childfree
P1	29	B.S.	Website Designer	5	2	Childfree
P2	35	B.S.	Project writer	8	8.5	Childfree
P3	33	M.S.	Teacher	10	9	Childfree
P4	37	Ph.D.	Academician	6	10	Childfree
P5	25	B.S.	Veterinarian	1	1	Childfree
P6	29	B.S.	Trainer	5	1.5	Childfree
P7	39	B.S.	Real estate agent	16	13	Childfree
P8	33	Ph.D.	Academician	10	3.5	Childfree
P9	43	Ph.D.	Academician	3	15	Childfree
P10	30	M.S.	Academician	4	3	Childfree
P11	32	B.S.	Police	10	4	Childfree
P12	38	B.S.	Teacher	12	12	Mother (2 children: 7/10)
P13	40	Ph.D.	Academician	18	7	Mother (1 child: 2.5)
P14	33	B.S.	Technician	15	8	Mother (2 children: 3/5)
P15	37	B.S.	Civil servant	6	11	Mother (1 child: 9)
P16	38	Ph.D.	Academician	7	11	Mother (1 child: 9)
P17	36	M.S.	Teacher	12	8	Mother (1 child: 7)
P18	42	B.S.	Business owner	14	14	Mother (1 child: 9)
P19	37	Ph.D.	Academician	13	13	Mother (2 children: 4/9)
P20	44	B.S.	Civil servant	25	23	Mother (2 children: 13/21)
P21	41	HS	Worker	13	16	Mother (1 child: 16)
P22	43	Ph.D.	Academician	16	21	Mother (2 children: 11/17)

**Table 3:** Marital and life satisfaction differences in childfree women and mothers.

Group	Marital Satisfaction		Life Satisfaction	
	*M*	*SD*	*M*	*SD*
Childfree women	53.35	9.84	21.61	6.09
Mothers	52.50	9.29	23.79	7.05

Note. *M*: Mean; SD: Standard Deviation.

**Table 4:** Multivariate and univariate analysis of variance *F* ratios for motherhood status for MS and SWLS.

Variable	MANOVA	ANOVA F(1,176)	
	F(2,175)	MS	SWLS
Motherhood status	2.241	0.211	2.547

Note. p<0.05.

## Data Availability

The dataset generated and analyzed during the current study is available from the corresponding author upon reasonable request.

## References

[1] Agrillo C., Nelini C. (2008). Childfree by choice: A review. J. Cult. Geogr..

[2] Attwood L., Isupova O., Attwood L., Schimpfössl E., Yusupova M. (2018). Choosing whether to have children: A netnographic study of women’s attitudes towards childbirth and the family in Post-Soviet Russia. Gender and Choice After Socialism.

[3] Blackstone A., Stewart M.D. (2012). Choosing to be childfree: Research on the decision not to parent. Sociol. Compass.

[4] Iwasawa M. (2004). Partnership transition in contemporary Japan: Prevalence of childless non-cohabiting couples. Jpn. J. Popul..

[5] Maher J.M., Saugeres L. (2007). To be or not to be a mother? Women negotiating cultural representations of mothering. J. Sociol..

[6] Martinez G., Daniels K., Chandra A. (2012). Fertility of men and women aged 15–44 years in the United States: National survey of family growth, 2006–2010. Natl. Health Stat. Rep..

[7] Miettinen A. (2010). Voluntary or involuntary childlessness? Socio-demographic factors and child-lessness intentions among childless Finnish men and women aged 25–44. Finn. Yearb. Popul. Res..

[8] Savelieva K., Jokela M., Rotkirch A. (2022). Reasons to postpone childbearing during fertility decline in Finland. Marriage Fam. Rev..

[9] Hara T. (2008). Increasing childlessness in Germany and Japan: Toward a childless society?. Int. J. Jpn. Sociol..

[10] Badinter E., Ekmekçi A. (2017). Kadınlık Mı? Annelik Mi? [The Conflict: Woman & Mother].

[11] Hird J.M., Abshoff K. (2000). Women without children: A contradiction in terms?. J. Comp. Fam. Stud..

[12] Moore J. (2014). Reconsidering childfreedom: A feminist exploration of discursive identity construction in childfree livejournal communities. Women’s Stud. Commun..

[13] Yeshua-Katz D. (2017). Childless in an IVF-nation: Online stigma-coping strategies in support groups for childless Israeli women. Inf. Commun. Soc..

[14] Bayraktar S. (2011). Makbul Anneler, Müstakbel Vatandaşlar: Neoliberal Beden Politikalarında Annelik [Acceptable Mothers, Prospective Citizens: Motherhood in Neoliberal Body Politics].

[15] Eke N.P. (2020). Discursive Construction of the Childfree Choice. Ph.D. Thesis.

[16] Parlak S., Tekin I. (2020). A phenomenological study on voluntarily childless women. Stud. Psychol..

[17] Meşe İ. (2014). Bir sorunsal olarak popüler yayınlarda “ideal annelik” kurgusu. Muhafazakâr Düşünce Derg..

[18] Beck U., Beck-Gernsheim E., Ermiş N. (2012). Aşkın Normal Kaosu [The Normal Chaos of Love].

[19] Gillespie R. (2003). Childfree and feminine: Understanding the gender identity of voluntarily childless women. Gend. Soc..

[20] Butler J. (1993). Bodies That Matter: On the Discursive Limits of Sex.

[21] OECD (2023). Family Database—SF2.4: Share of Births Outside Marriage.

[22] Feldman H. (1981). A comparison of intentional parents and intentionally childless couples. J. Marriage Fam..

[23] Rubin-Terrado M.A. (1994). Social Supports and Life Satisfaction of Older Childless Women and Mothers Living in Nursing Homes. Ph.D. Thesis.

[24] Somers M.D. (1993). A comparison of voluntarily childfree adults and parents. J. Marriage Fam..

[25] Donath O. (2015). Regretting motherhood: A sociopolitical analysis. J. Women Cult. Soc..

[26] Donath O., Baştimur B.Y. (2018). Abartılmış Annelik Bastırılmış Kadınlık [Regretting Motherhood].

[27] Rich A. (1976). Of Woman Born: Motherhood as Experience and Institution.

[28] Bell K. (2013). Constructions of “infertility” and some lived experiences of involuntary childlessness. Affilia.

[29] Hansen T. (2012). Parenthood and happiness: A review of folk theories versus empirical evidence. Soc. Indic. Res..

[30] Huijts T., Kraaykamp G., Subramanian S.V. (2013). Childlessness and psychological well-being in context: A multilevel study on 24 European countries. Eur. Sociol. Rev..

[31] Jeffries S., Konnert C. (2002). Regret and psychological well-being among voluntarily and involuntarily childless women and mothers. Int. J. Aging Hum. Dev..

[32] Peterson H. (2015). Fifty shades of freedom. Voluntary childlessness as women’s ultimate liberation. Women’s Stud. Int. Forum.

[33] Cwikel J., Gramotnev H., Lee C. (2006). Never-married childless women in Australia: Health and social circumstances in older age. Soc. Sci. Med..

[34] Keizer R., Dykstra P.A., Jansen M.D. (2008). Pathways into childlessness: Evidence of gendered life course dynamics. J. Biosoc. Sci..

[35] Hakim C. (2000). Work-Lifestyle Choices in the 21st Century: Preference Theory.

[36] McAllister F., Clarke L. (1998). Choosing Childlessness.

[37] Rempel J. (1985). Childless elderly: What are they missing?. J. Marriage Fam..

[38] Callan V.J. (1987). The personal and marital adjustment of mothers and of voluntarily and involuntarily childless wives. J. Marriage Fam..

[39] Hutchinson R. (2022). ‘I try to be true to myself but other people have to fit you into a box’: Exploring how voluntarily childfree women discursively negotiate their sense of self in a pronatalist society. Psychol. Women Equal. Rev..

[40] Mollen D. (2006). Voluntarily childfree women: Experiences and counseling considerations. J. Ment. Health Couns..

[41] Bem S.L. (1981). Gender schema theory: A cognitive account of sex typing. Psychol. Rev..

[42] Thornham S., Gamble S. (2001). Second wave feminism. Feminism and Postfeminism.

[43] Creswell J.W., Plano Clark V.L. (2011). Designing and Conducting Mixed Methods Research.

[44] Neubauer B.E., Witkop C.T., Varpio L. (2019). How phenomenology can help us learn from the experiences of others. Perspect. Med. Educ..

[45] Creswell J.W., Creswell J.D. (2017). Research Design: Qualitative, Quantitative, and Mixed Methods Approaches.

[46] Gentles S.J., Charles C., Ploeg J., McKibbon K. (2015). Sampling in qualitative research: Insights from an overview of the methods literature. The Qualitative Report.

[47] Büyüköztürk Ş. (2002). Sosyal Bilimler Için Veri Analizi el Kitabı [Data Analysis Handbook for the Social Sciences].

[48] Tabachnick B.G., Fidell L.S. (2012). Using Multivariate Statistics.

[49] Çelik M., Yazgan İnanç B. (2009). Evlilik doyum ölçeği: Geçerlik ve güvenirlik çalışmaları [Marriage satisfaction scale: Validity and reliability studies]. Sos. Bilim. Enst. Derg..

[50] Diener E.D., Emmons R.A., Larsen R.J., Griffin S. (1985). The satisfaction with life scale. J. Personal. Assess..

[51] Durak M., Şenol-Durak E., Gençöz T. (2010). Psychometric properties of the satisfaction with life scale among Turkish university students, correctional officers, and elderly adults. Soc. Indic. Res..

[52] Roesad M., Rumondor P. (2021). Happily married in the absence of a child: Marital satisfaction of voluntary and involuntary childless individuals. Emerging Issues in Humanity Studies and Social Sciences (ICE-HUMS 2021).

[53] Shenaar-Golan V., Lans O. (2022). Measuring differentiation of self to evaluate subjective well-being in women who are childfree by choice. Fam. J..

[54] Watling Neal J., Neal Z.P. (2012). Prevalence and characteristics of childfree adults in Michigan (USA). PLoS ONE.

[55] Hansen T., Slagsvold B., Moum T. (2009). Childlessness and psychological well-being in midlife and old age: An examination of parental status effects across a range of outcomes. Soc. Indic. Res..

[56] Bien A., Rzonca E., Iwanowicz-Palus G., Lecyk U., Bojar I. (2017). Quality of life and satisfaction with life of women who are childless by choice. Ann. Agric. Environ. Med..

[57] Dykstra P.A., Wagner M. (2007). Pathways to childlessness and late-life outcomes. J. Fam. Issues.

[58] Stahnke B., Blackstone A., Howard H. (2020). Lived experiences and life satisfaction of childfree women in late life. Fam. J..

[59] Stahnke B., Cooley M.E., Blackstone A. (2022). A systematic review of life satisfaction experiences among childfree adults. Fam. J..

[60] Zhang W., Liu G. (2007). Childlessness, psychological well-being, and life satisfaction among the elderly in China. J. Cross-Cult. Gerontol..

[61] Stanca L. (2012). Suffer the little children: Measuring the effects of parenthood on well-being worldwide. J. Econ. Behav. Organ..

[62] Szcześniak M., Świątek B., Słowik A. (2019). Big five personality traits and life satisfaction. Religions.

[63] Gobbi P.E. (2013). A model of voluntary childlessness. J. Popul. Econ..

[64] Miller T., Tunçer G. (2010). Annelik Duygusu: Mitler ve Deneyimler [Making Sense of Motherhood: A Narrative Approach].

[65] Ramu G.N. (1984). Family background and perceived marital happiness: A comparison of voluntary childless couples and parents. Can. J. Sociol..

[66] Schmid W., Bora T. (2018). Anne Baba ve Büyükanne Büyükbaba Olmanın Sevinçleri Üzerine [On the Joy of Being a Parent and Grandparent].

[67] Sayın Ö. (2020). Aile Sosyolojisi [Sociology of Family].

[68] Glenn E.N., Chang G., Forcey L.R. (1994). Mothering: Ideology, Experience, and Agency.

[69] Douglas S., Michaels M. (2004). The Mommy Myth.

[70] Beauvoir S., Onaran B. (1976). İkinci Cins [The Second Sex].

[71] Eliuz Ü. (2011). Cinsel kimlik paniği: Kadın olmak. Turk. Stud..

[72] Friedan B. (1963). The Feminine Mystique.

[73] Phoenix A., Woollett A.T., Phoenix A., Woollett A., Lloyd E. (1991). Motherhood, social construction, politics and psychology. Motherhood: Meanings, Practices and Ideologies.

[74] Gittins D., Erdem T. (1985). Aile Sorgulanıyor [The Family in Question].

[75] Koropeckyj-Cox T., Romano V., Moras A. (2007). Through the lenses of gender, race, and class: Students’ perceptions of childless/childfree individuals and couples. Sex Roles.

[76] Park K. (2002). Stigma management among the voluntarily childless. Sociol. Perspect..

